# Improving contig binning of metagenomic data using ${d}_2^S$ oligonucleotide frequency dissimilarity

**DOI:** 10.1186/s12859-017-1835-1

**Published:** 2017-09-20

**Authors:** Ying Wang, Kun Wang, Yang Young Lu, Fengzhu Sun

**Affiliations:** 1 Department of Automation, Xiamen University, Xiamen, Fujian 361005 China; 2 Molecular and Computational Biology Program, University of Southern California, Los Angeles, California, CA 90089 USA; 3 Center for Computational Systems Biology, Fudan University, Shanghai, 200433 China

**Keywords:** Metagenomics, Contig binning, Taxonomy-independent, ${d}_2^S$ dissimilarity, *k*-tuple

## Abstract

**Background:**

Metagenomics sequencing provides deep insights into microbial communities. To investigate their taxonomic structure, binning assembled contigs into discrete clusters is critical. Many binning algorithms have been developed, but their performance is not always satisfactory, especially for complex microbial communities, calling for further development.

**Results:**

According to previous studies, relative sequence compositions are similar across different regions of the same genome, but they differ between distinct genomes. Generally, current tools have used the normalized frequency of *k*-tuples directly, but this represents an absolute, not relative, sequence composition. Therefore, we attempted to model contigs using relative *k*-tuple composition, followed by measuring dissimilarity between contigs using ${d}_2^S$. The ${d}_2^S$ was designed to measure the dissimilarity between two long sequences or Next-Generation Sequencing data with the Markov models of the background genomes. This method was effective in revealing group and gradient relationships between genomes, metagenomes and metatranscriptomes. With many binning tools available, we do not try to bin contigs from scratch. Instead, we developed ${d}_2^S\mathrm{Bin}$ to adjust contigs among bins based on the output of existing binning tools for a single metagenomic sample. The tool is taxonomy-free and depends only on *k*-tuples. To evaluate the performance of ${d}_2^S\mathrm{Bin}$, five widely used binning tools with different strategies of sequence composition or the hybrid of sequence composition and abundance were selected to bin six synthetic and real datasets, after which ${d}_2^S\mathrm{Bin}$ was applied to adjust the binning results. Our experiments showed that ${d}_2^S\mathrm{Bin}$ consistently achieves the best performance with tuple length *k* = 6 under the independent identically distributed (i.i.d.) background model. Using the metrics of *recall*, *precision* and *ARI* (Adjusted Rand Index), ${d}_2^S\mathrm{Bin}$ improves the binning performance in 28 out of 30 testing experiments (6 datasets with 5 binning tools). The ${d}_2^S\mathrm{Bin}$ is available at https://github.com/kunWangkun/d2SBin.

**Conclusions:**

Experiments showed that ${d}_2^S$ accurately measures the dissimilarity between contigs of metagenomic reads and that relative sequence composition is more reasonable to bin the contigs. The ${d}_2^S\mathrm{Bin}$ can be applied to any existing contig-binning tools for single metagenomic samples to obtain better binning results.

**Electronic supplementary material:**

The online version of this article (10.1186/s12859-017-1835-1) contains supplementary material, which is available to authorized users.

## Background

Metagenomics sequencing provides deep insights into microbial communities [1]. A key step toward investigating their taxonomic structure within metagenomics data involves assigning assembled contigs into discrete clusters known as bins [2]. These bins represent species, genera or higher taxonomic groups [3]. Therefore, efficient and accurate binning of contigs is essential for metagenomics studies.

The binning of contigs remains challenging owing to repetitive sequence regions within or across genomes, sequencing errors, and strain-level variation within the same species [4]. Many studies have reported on binning, essentially highlighting two different strategies [5]: “taxonomy-dependent” supervised classification and “taxonomy-independent” unsupervised clustering. “Taxonomy-dependent” studies are based on sequence alignments [6], phylogenetic models [7, 8] or oligonucleotide patterns [9]. “Taxonomy-independent” studies extract features from contigs to infer bins based on sequence composition [10–14], abundance [15], or hybrids of both sequence composition and abundance [4, 5, 16–18]. Therefore, these approaches can be applied to bin contigs from incomplete or uncultivated genomes. Some hybrid binning tools, such as COCACOLA [5], CONCOCT [4], MaxBin2.0 [18] and GroopM [16], are designed to bin contigs based on multiple related metagenomic samples. Contigs with similar coverage profiles are more likely to come from the same genome. Previous studies showed that co-varying coverage profiles across multiple related metagenomes play important roles in contig binning [4, 5]. The multiple related samples should be temporal or spatial samples of a given ecosystem [16] composed of similar microbial organisms, but different abundance levels. However, in many situations, multiple related samples may not be available in the required numbers, and as a result, contig-binning based on single metagenomes is still important.

Contig binning tools based on a single sample generally follow one of three strategies. 1) Sequence composition. It is usually denoted as frequencies of *k*-tuples (*k*-mers) with *k*= 2–6 as genomic signatures of contigs. MetaWatt [12] and SCIMM [11] built multivariate statistics and/or interpolated Markov models of background genomes to bin the contigs. Metacluster 3.0 [14] clustered the contigs using *k*-tuple frequency and Spearman correlation between the *k*-tuple frequency vectors. LikelyBin [10] utilized Markov Chain Monte Carlo approaches based on 2- to 5-tuples. 2) Abundance. AbundanceBin [15] estimated the relative abundance levels of species living in the same environment based on Poisson distributions of 20-tuples with an Expectation Maximization (EM) algorithm. The MBBC [19] package estimated the abundance of each genome using the Poisson process. All tools based on abundance are designed to bin short or long reads instead of assembled contigs. 3) Hybrid of composition and abundance. Maxbin1.0 [17] combined *4*-tuple frequencies and scaffold coverage levels to populate the genomic bins using single-copy marker genes and an Expectation Maximization (EM) algorithm. MyCC [20] combined genomic signatures, marker genes and optional contig coverages within one or multiple samples.

Contig binning using *k*-tuple composition is based on the observation that relative sequence compositions are similar across different regions of the same genome, but differ between distinct genomes [21, 22]. The frequency vector of *k*-tuples is one of the representation of sequence composition. In general, current tools use the frequency of *k*-tuples directly, but this represents absolute, not relative, sequence composition. Here, “absolute” frequency refers to the number of occurrences of a *k*-tuple over the total number of occurrences of all *k*-tuples. On the other hand, “relative” frequency refers to the difference between the observed frequency of a *k*-tuple and the corresponding expected frequency under a given background model. Contigs in the same bin are from the same taxonomic group, such as one class, species or strain. Therefore, contigs from the same bin are expected to obey a consistent background model. Several sequence dissimilarity measures based on relative frequencies of *k*-tuples have been developed such as CVTree, $$ {d}_2^{\ast } $$ and $$ {d}_2^S, $$ and recent studies [23–27] have shown that $$ {d}_2^S $$ is superior to other dissimilarity measures for the comparison of genome sequences based on relative *k*-tuple frequencies. Therefore, in the present study, we attempted to model the relative sequence composition and measure dissimilarity between contigs with $$ {d}_2^S $$ for a single metagenomic sample. The $$ {d}_2^S $$ was designed to measure the dissimilarity between two sequences or next generation sequencing data by modeling the background genomes [23] using Markov and interpolated Markov chains. Previous studies verified the effectiveness of $$ {d}_2^S $$ in revealing group and gradient relationships between genomes [24, 25], metagenomes [28] and metatranscriptomes [26, 27]. However, binning of contigs directly using $$ {d}_2^S $$ is computationally expensive and impractical for large metagenomics studies due to the need to construct Markov background models for sequences and to calculate the expected counts of *k*-tuples. On the other hand, many binning tools based on absolute *k*-tuple frequencies and the results from such methods are reasonable. Still, these tools and methods can be improved by using $$ {d}_2^S $$ dissimilarity. Therefore, in the present study, we do not bin the contigs from scratch. Instead, we attempt to adjust contig bins based on the output of any existing binning tools. We model each contig with a Markov chain based on its *k*-tuple frequency vector. The bin’s center is represented by the averaged *k*-tuple frequency vectors of all contigs in this bin and is also modeled with a Markov chain. Then, $$ {d}_2^S $$ measures dissimilarity between a contig and a bin’s center based on relative sequence composition, as represented by the Markov chains. Finally, a K-means clustering algorithm is applied to cluster the contigs based on the $$ {d}_2^S $$ dissimilarities, where K is the number of clusters. Such an approach, on the one hand, overcomes the issue of extensive computational complexity directly using $$ {d}_2^S $$ and, on the other hand, further improves the initial binning results. The method is developed as an open source package, termed $$ {d}_2^S\mathrm{Bin} $$, which is available at https://github.com/kunWangkun/d2SBin.

We selected six synthetic and real datasets that had originally been used to evaluate existing tools as testing datasets. ${d}_2^S\mathrm{Bin}$ was applied to adjust the binning results of five representative binning tools using sequence composition (MetaCluster3.0 [14], MetaWatt [12] and SCIMM [11]) and the hybrid of sequence composition and abundance (MaxBin1.0 [17], MyCC [20]) based on a single metagenomic sample. Tuple length *k* = 6 and the independent identically distributed (i.i.d.) background model (i.e., Markov order *r* = 0) are frequently the optimal parameters for ${d}_2^S\mathrm{Bin}$ to achieve the best performance for metagenomics contig binning. ${d}_2^S\mathrm{Bin}$ improved the binning results in 28 out of 30 testing experiments for 6 datasets using 5 binning tools, giving significantly better performance in terms of *recall*, *precision* and *ARI* (Adjusted Rand Index).

## Methods

**Fig. 1:**
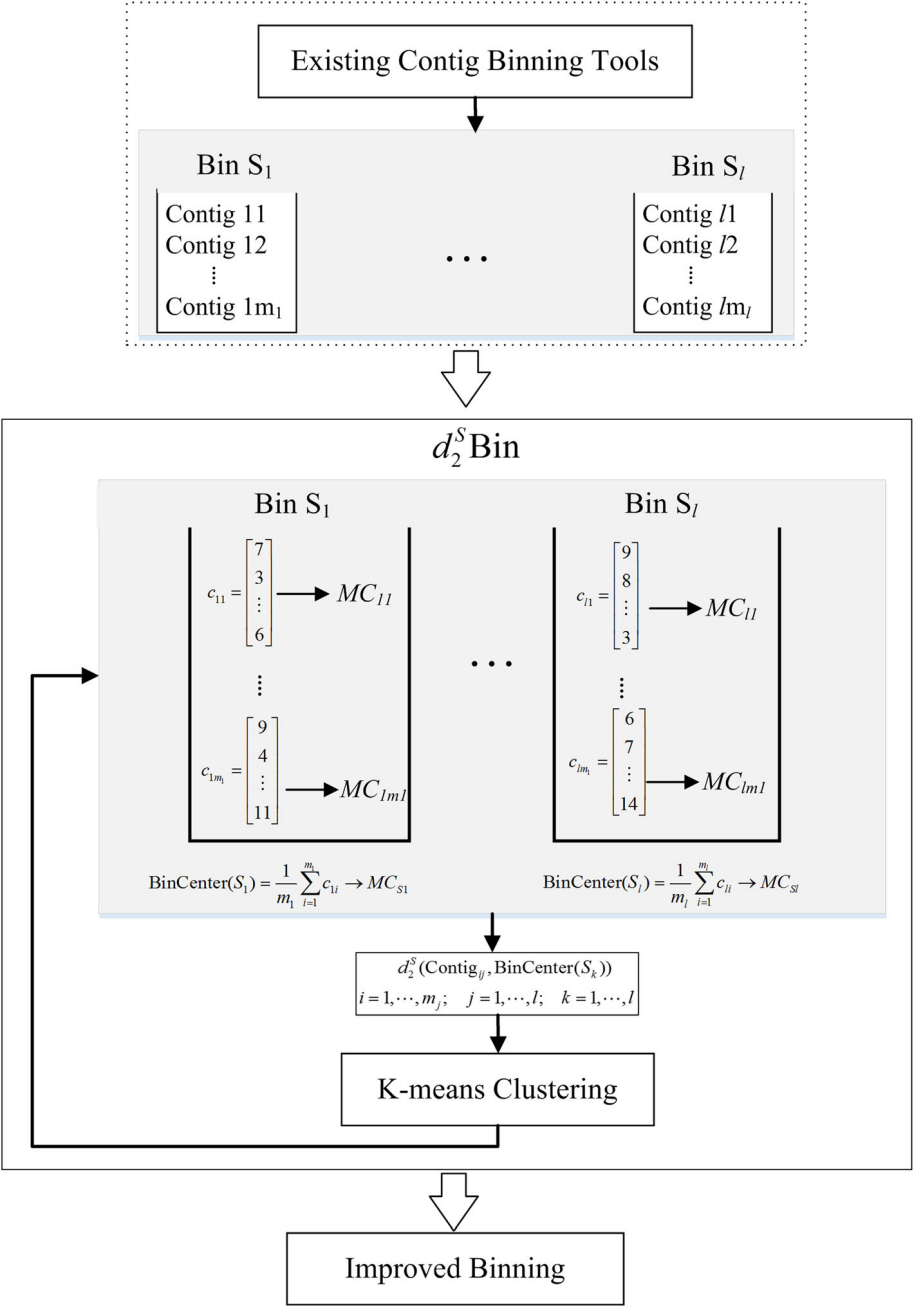
Flowchart contig binning with ${d}_2^S\mathrm{Bin}$

The framework of ${d}_2^S\mathrm{Bin}$ is shown in the flowchart of Fig. 1. Any existing contig binning tool is applied with its default settings to bin the contigs in a single metagenomic sample. Each contig is modeled with a Markov chain based on its *k*-tuple frequency vector. For each bin, the bin’s center is also modeled with a Markov chain based on the averaged frequency vector of all contigs in this bin. The ${d}_2^S$ measures the dissimilarity between a contig and a bin’s center based on the background probability models. Assuming that contigs in the same bin come from an identical background model, the ${d}_2^S$ dissimilarity between contigs from the same bin should be smaller than that between contigs from different bins under correct binning. The K-means algorithm is then applied to adjust the contigs among different bins to minimize the within-bin sum of squares based on ${d}_2^S$ dissimilarity.

### The ${d}_2^S$ dissimilarity measure between two contigs based on *k*-tuple sequence signature

The ${d}_2^S$ is a normalized dissimilarity measure for two sequences based on either long genomic sequences or NGS short reads in which expected word counts are subtracted from the observed counts for each sequence. The background adjusted word counts are then compared using correlation to measure the dissimilarity between the two sequences [25]. Let ${c}_X=\left({c}_{X,1},{c}_{X,2},\cdots, {c}_{X,{4}^k}\right)$ and ${c}_Y=\left({c}_{Y,1},{c}_{Y,2},\cdots, {c}_{Y,{4}^k}\right)$ be the *k-*tuple frequency vectors from two sequences *X* and *Y*, respectively, where *c*_*X* , *i*_ is the occurring times of the *i* ^th^ *k*-tuple in sequence *X* and *i* = 1 ⋯ 4^*k*^. At each base in the tuple, there are four possible nucleotides, that is A, C, G, and T, for nucleotide sequences. So there are 4^*k*^ combinations when tuple length is *k*.

The ${d}_2^S$ dissimilarity is defined as

1 $$ {d}_2^S\left({\tilde{c}}_X,{\tilde{c}}_Y\right)=\frac{1}{2}\left(1-\frac{D_2^S\left({\tilde{c}}_X,,,{\tilde{c}}_Y\right)}{\sqrt{\sum_{i=1}^{4^k}\frac{{\tilde{c}}_{X,i}^2}{\sqrt{{\tilde{c}}_{X,i}^2+{\tilde{c}}_{Y,i}^2}}}\sqrt{\sum_{i=1}^{4^k}\frac{{\tilde{c}}_{Y,i}^2}{\sqrt{{\tilde{c}}_{X,i}^2+{\tilde{c}}_{Y,i}^2}}}}\right), $$

where

2 $$ {D}_2^S\left({\tilde{c}}_X,{\tilde{c}}_Y\right)=\sum_{i=1}^{4^k}\frac{{\tilde{c}}_{X,i}{\tilde{c}}_{Y,i}}{\sqrt{{\tilde{c}}_{X,i}^2+{\tilde{c}}_{Y,i}^2}}, $$

3 $$ {\tilde{c}}_{X,i}={c}_{X,i}-{n}_X{p}_{X,i},\kern0.5em {\tilde{c}}_{Y,i}={c}_{Y,i}-{n}_Y{p}_{Y,i}, $$

where *p*_• , *i*_ is the probability of the *i* ^*th*^ *k-*tuple under the Markov model with order *r* = 0 − 3 for one long sequence or set of reads and ${n}_{\bullet }=\sum_{i=1}^{4^k}{c}_{\bullet, i}$, • = *X* or *Y* is the sum of occurrences of all *k-*tuples. The value of ${d}_2^S$ is between 0 and 1. The *p*_*X* , *i*_ is the probability of the *i* ^*th*^ *k-*tuple under the background sequence for *X*. The *p*_*X* , *i*_ can be the probability under the i.i.d. model, or under the Markov chain of different orders. The *i* ^*th*^ *k-*tuple is denoted as *w* = *w*_1_ *w*_2⋯_ *w*_*k*_. Under the *r* ^*th*^ order Markov chain *M*_*r*_, the probability of the *k*-tuple *w*, namely the expected frequency, can be computed as

4 $$ p\left(w|{M}_r\right)=\left\{\begin{array}{l}\prod \limits_{j=1}^kp\left({w}_j\right)\kern5.00em r=0\\ {}p\left({w}_1{w}_2\dots {w}_r\right)\prod \limits_{j=1}^{k-r}p\left({w}_{j+r}|{w}_j{w}_{j+1}\dots {w}_{j+r-1}\right)\kern0.5em 1\le r\le k-1\end{array}\right. $$

where *p*(*w*_*j*_) is the probability of *w*_*j*_ estimated by the ratio of the number of occurrences of *w*_*j*_ over the number of all nucleotides. The value of *p*(*w*_1_ *w*_2⋯_ *w*_*r*_) is estimated by the ratio of the number of occurrences of *w*_1_ *w*_2⋯_ *w*_*r*_ over all the number of *r*-tuple occurrences. The value of *p*(*w*_*j* + *r*_| *w*_*j*_ *w*_*j* + 1⋯_ *w*_*j* + *r* − 1_) is estimated by the fraction of occurrences of *w*_*j* + *r*_ conditional on the previous occurrences of *w*_*j*_ *w*_*j* + 1⋯_ *w*_*j* + *r* − 1_.

### ${d}_2^S\mathrm{Bin}$: Contig binning based on the ${d}_2^S$ measure

Let *S* = {*S*_1_, *S*_2_, ⋯*S*_*l*_} be the partition of all contigs into *l* bins. Contig *X* is represented as ${c}_X=\left({c}_{X,1},{c}_{X,2},\cdots, {c}_{X,{4}^k}\right)$, the occurrence vector of *k-*tuples within the contig. The center of bin *S*_*j*_ is represented as the average frequency vector,

5 $$ {c}_{S_j}=\frac{1}{n_j}{\sum}_{X_i\in {S}_j}{C}_{X_i}, $$

where *X*_*i*_ is the contig currently in *S*_*j*_ and *n*_*j*_ is the number of contigs in *S*_*j*_. The value of ${d}_2^S\left({\overset{\sim }{c}}_X,{\overset{\sim }{c}}_{S_j}\right)$ quantifies the dissimilarity between contig *X* and bin *S*_*j*_.

In our study, when the number of bins is fixed, the metrics of binning call for minimizing the within-bin sum of squares based on ${d}_2^S$ dissimilarity, that is,

6 $$ \underset{s}{\arg \min}\sum_{j=1}^l\sum_{X\in {S}_j}{d}_2^s\left({\tilde{c}}_X,{\tilde{c}}_{S_j}\right). $$

We then used the K-means clustering algorithm to optimize Eq. (6).

### Experimental design

The purpose of our study is to improve binning results using ${d}_2^S\mathrm{Bin}$ based on the output of current existing binning tools. Therefore, we adopted both synthetic and real testing datasets generated, or used, by previous binning tools in order to test the performance of ${d}_2^S\mathrm{Bin}$, as shown in Table 1. The ${d}_2^S\mathrm{Bin}$ was applied to the binning results of five contig-binning tools, respectively, to evaluate its performance in improving their binning results.

**Table 1:** Synthetic and real testing datasets for contig binning

Testing datasets		Tools tested previously	Tools tested in this study	
Synthetic	*10 genomes: 20×* *10 genomes: 80×*	Maxbin 1.0 [17]	MaxBin 1.0 [17]	+${d}_2^S\mathrm{Bin}$
			MetaCluster 3.0 [14]	
	*100 genomes:simLC+* *100 genomes:simMC+* *100 genomes:simHC+*		MetaWatt3.5.3 [3]	
			SCIMM 0.3.0 [11]	
Real	*Sharon*	COCACOLA [5] CONCOCT [4]		
			MyCC_2017 [20]	

#### Selection of contig binning tools

The ${d}_2^S\mathrm{Bin}$ was applied to adjust the contig-binning results from MaxBin1.0 [17], MetaCluster3.0 [14], MetaWatt [12], MyCC [20] and SCIMM [11] to evaluate its performance. These five widely used contig-binning tools use different binning strategies to bin the contigs for single metagenomic sample: 1) Sequence composition: MetaCluster3.0 [14] measures the Spearman distance between *4*-tuple frequency vectors and bins contigs with the K-median algorithm. The MetaCluster4.0 [29] and 5.0 [30] were designed to bin the reads from metagenomics samples of different abundance characteristics. MetaWatt [12] and SCIMM [11] build interpolated Markov models of the background genomes and assign the contigs to bins with maximum likelihood. 2) Hybrid of abundance and sequence composition: MaxBin1.0 [17] measures the Euclidean distance between *4*-tuple frequency vectors of contigs and assigns them with an EM algorithm, taking scaffold coverage levels into consideration. MyCC [20] combines genomic signatures, marker genes and optional contig coverages within one or multiple samples.

#### Five synthetic testing datasets with 10 genomes and 100 genomes

MaxBin1.0 [17] used these five datasets to evaluate its performance. Here we used the same five datasets to evaluate the performance of ${d}_2^S\mathrm{Bin}.$ Short reads were simulated by MetaSim [31] and assembled to contigs by Velvet [32]. The contigs and their labels are available for downloading from the MaxBin1.0 paper [17]. For the metagenomes containing 10 genomes, 5 million and 20 million paired-end reads were sampled as *20×* and *80×* average coverage, respectively. For the metagenomes containing 100 genomes, 100 million paired-end reads were sampled with three settings to create *simLC+*, *simMC+* and *simHC+*. The three datasets represent microbial communities with different levels of complexity, which mimicked the setting of the previous study [33]: *simLC* simulates low-complexity communities dominated by a single near-clonal population flanked by low-abundance ones. Such datasets result in a near-complete draft assembly of the dominant population in, for example, bioreactor communities [34]. *simMC* resembles moderately complex communities with more than one dominant population, also flanked by low-abundance ones, as has been observed in an acid mine drainage biofilm [35] and *Olavius algarvensis* symbionts [36]. These types of communities usually result in substantial assembly of the dominant populations according to their clonality. *simHC* simulates high-complexity communities lacking dominant populations, such as agricultural soil [37], where no dominant strains are present and minimal assembly results. In addition, the empirical 80-bps error model, which incorporates different error types (deletion, insertion, substitution) at certain positions with empirical error probabilities for Illumina, was produced by MetaSim [31] and used in simulating all metagenomes [17].

#### One real testing dataset, *Sharon*

This dataset was applied to test the binning tools COCACOLA [5] and CONCOCT [4]. The dataset is composed of a time-series of 11 fecal microbiome samples from a premature infant [38], denoted as ‘*Sharon*’. All metagenomic sequencing reads from the 11 samples were merged together, and 5579 contigs were assembled. The contigs were annotated with TAXAassign [39], and 2614 contigs were unambiguously aligned to 21 species [5].

The above datasets cover various species diversity, species dissimilarity, sequencing depth, and community complexity. They include synthetic and real data. Therefore, testing on these datasets would yield a comprehensive evaluation of ${d}_2^S\mathrm{Bin}$.

### Evaluation criteria

To evaluate the performance of ${d}_2^S\mathrm{Bin}$, three commonly used criteria in binning studies [4, 5, 17], *recall*, *precision* and *ARI* (Adjusted Rand Index), were applied in our study. As described in COCACOLA [5], the binning result is represented as a *K* × *S* matrix *A* = (*a*_*ks*_) with *K* bins on *S* species where *a*_*ks*_ indicates the shared number of contigs between the *k* ^*th*^ bin and the *s* ^*th*^ species. Each contig binning tool filters out low-quality contigs; therefore, *N* is the total number of contigs passing through the filter and binned by the tools.

*Recall*: For each species, we first find the bin that contains the maximum number of contigs from the species. We then sum over the maximum number of all species and divide by the number of contigs.

7 $$ recall=\frac{1}{N}{\sum}_s{max}_k\left\{{a}_{ks}\right\} $$

*Precision*: For each contig bin, we first find the species with the maximum number of contigs assigned to the bin. We then sum the maximum numbers across all bins and divide by the number of contigs.

8 $$ precision=\frac{1}{N}{\sum}_k{max}_s\left\{{a}_{ks}\right\} $$

*ARI* (Adjusted Rand Index): *ARI* is a unified measure of clustering results to determine how far from that perfect grouping a bin result falls. *ARI* focuses on whether pairs of contigs belonging to the same species can be binned together or not. The detailed descriptions can be found in [4, 5].

9 $$ ARI=\frac{\sum_{k,s}\left(\begin{array}{c}{a}_{ks}\\ {}2\end{array}\right)-{t}_3}{\frac{1}{2}\left({t}_1+{t}_2\right)-{t}_3} $$

where $$ {t}_1=\sum_k\left(\begin{array}{c}{a}_{k\bullet}\\ {}2\end{array}\right) $$, $$ {t}_2=\sum_s\left(\begin{array}{c}{a}_{\bullet s}\\ {}2\end{array}\right) $$, $$ {t}_3=\frac{2{t}_1{t}_2}{\left(\begin{array}{c}N\\ {}2\end{array}\right)}\kern0.5em $$ and *a*_*k*∙_ = ∑_*s*_ *a*_*ks*_, *a*_∙*s*_ = ∑_*k*_ *a*_*ks*_ .

## Results

In the calculation of ${d}_2^S$ dissimilarity, the setting of tuple length for *k*-tuple and Markov order for the background sequences are required. Based on previous studies [4, 5], for ${d}_2^S$, tuple length *k* was generally set to 4–7 tuples, and the order of Markov chain was generally set as 0–2, as in previous applications, to analyze metagenomic and metatranscriptomic samples [25, 26]. Therefore, we extended the testing range of tuple length and Markov order as 4–8 and 0–3 to assess the effect of tuple length and Markov order for ${d}_2^S\mathrm{Bin}$ on contig binning. As shown in Table 2, for the binning results of MaxBin on 10genome-80×, the i.i.d. (that is 0-order Markov) model obtained the highest three indexes at almost all tuple lengths. The models based on tuple length *k* = 6 represent superior performance. The best performance was achieved under the i.i.d. background model of 6-tuples. All three criteria dropped suddenly at *k* = 8. The experiment offered initial guidance for the selection of tuple length and Markov order.

**Table 2:** Initial assessments of the effects of tuple length and Markov order of the background sequences on the performance of MaxBin+ ${d}_2^S\mathrm{Bin}$ in terms of *recall*, *precision* and *ARI* for dataset *10genome-80×*

*10genome-80×*			Recall(%)	Precision(%)	ARI(%)
MaxBin			93.48	93.48	90.96
MaxBin+${d}_2^S\mathrm{Bin}$	k = 4	*r* = 0	**96.42**	**96.42**	**95.57**
		*r* = 1	93.99	93.99	90.86
		*r* = 2	86.35	86.35	76.18
	k = 5	r = 0	**96.83**	**96.83**	**96.03**
		r = 1	95.40	95.40	93.19
		r = 2	92.53	92.53	87.72
		*r* = 3	59.71	60.91	37.18
	k = 6	r = 0	***96.93***	***96.93***	***96.05***
		r = 1	96.01	96.01	94.57
		r = 2	94.24	94.24	91.40
		r = 3	81.28	83.77	71.56
	k = 7	r = 0	**94.41**	**94.41**	**92.08**
		r = 1	93.26	93.26	91.92
		r = 2	92.42	92.42	90.67
		r = 3	65.88	77.73	50.48
	k = 8	r = 0	**88.26**	82.94	80.04
		r = 1	87.17	**88.09**	**84.78**
		r = 2	87.19	87.12	82.73
		r = 3	60.08	73.08	46.46

The optimal numbers with respect Markov order are in bold

### Length selection of *k*-tuple in ${d}_2^S\mathrm{Bin}$

**Fig. 2:**
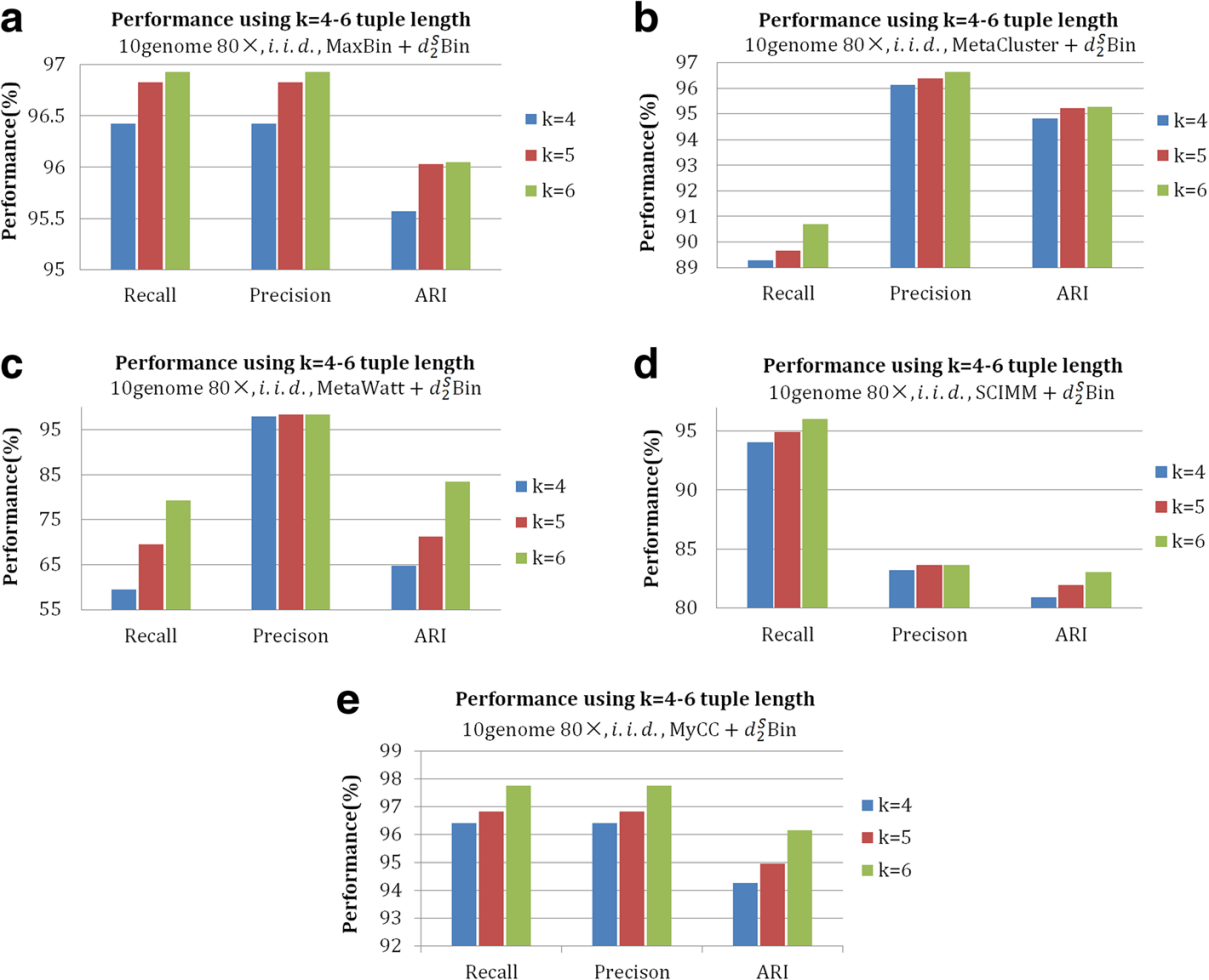
The effect of tuple length on the binning of contigs with different binning algorithms (MaxBin, MaxCluster, MetaWatt, SCIMM and MyCC) further modified by ${d}_2^S\mathrm{Bin}$ under the i.i.d. background model for dataset *10genome 80×*. **a**-**e** are the *Recall*, *Precision* and *ARI* of 4–6 tuples ${d}_2^S\mathrm{Bin}$ on the five contig-binning tools. From the figures, it can be clearly seen that 6-tuple ${d}_2^S\mathrm{Bin}$ achieves the best performance in almost all cases

**Fig. 3:**
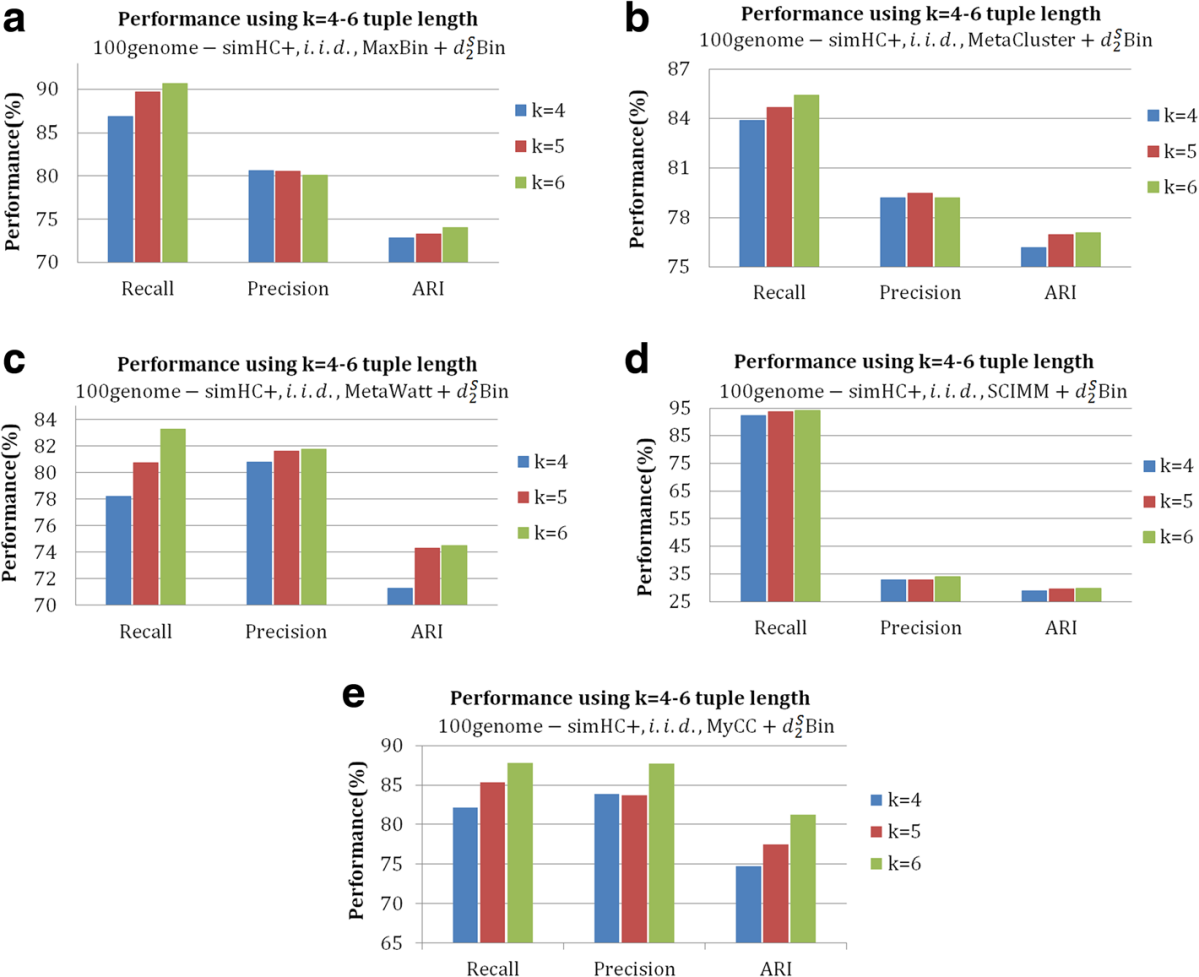
The effect of tuple length on the binning of contigs with different binning algorithms (MaxBin, MaxCluster, MetaWatt, SCIMM and MyCC) further modified by ${d}_2^S\mathrm{Bin}$ under the i.i.d. background model for dataset *100genome simHC+*. **a**-**e** are the *Recall*, *Precision* and *ARI* of 4–6 tuples ${d}_2^S\mathrm{Bin}$ on the five contig-binning tools*.* From the figures, it can be clearly seen that 6-tuple ${d}_2^S\mathrm{Bin}$ achieves the best performance in almost all cases

According to Table 2, we calculated ${d}_2^S$ with 4-8 bp tuples under the i.i.d. model based on the output of the existing binning tools. These tools were run under their default tuple length and mode. The datasets *10genome 80×* and *100genome-simHC+* were selected to test the effect of tuple length on the performance of ${d}_2^S\mathrm{Bin}$. For both datasets, ${d}_2^S\mathrm{Bin}$ based on 6-tuples achieved the best performance on *precision*, *recall* and *ARI* for all five tools. Figures 2 and 3 only plot the curves of tuple length *k* = 4–6 because the severe dropping in performance with *k* = 7, 8 led to an excessively wide Y-axis coordinate range, and the curves of *k* = 4–6 appeared to aggregate, making it hard to display the superiority of *k* = 6. Therefore, we set *k* = 6 with ${d}_2^S$ in the rest of our study.

### Order selection for Markov chain in ${d}_2^S\mathrm{Bin}$

**Fig. 4:**
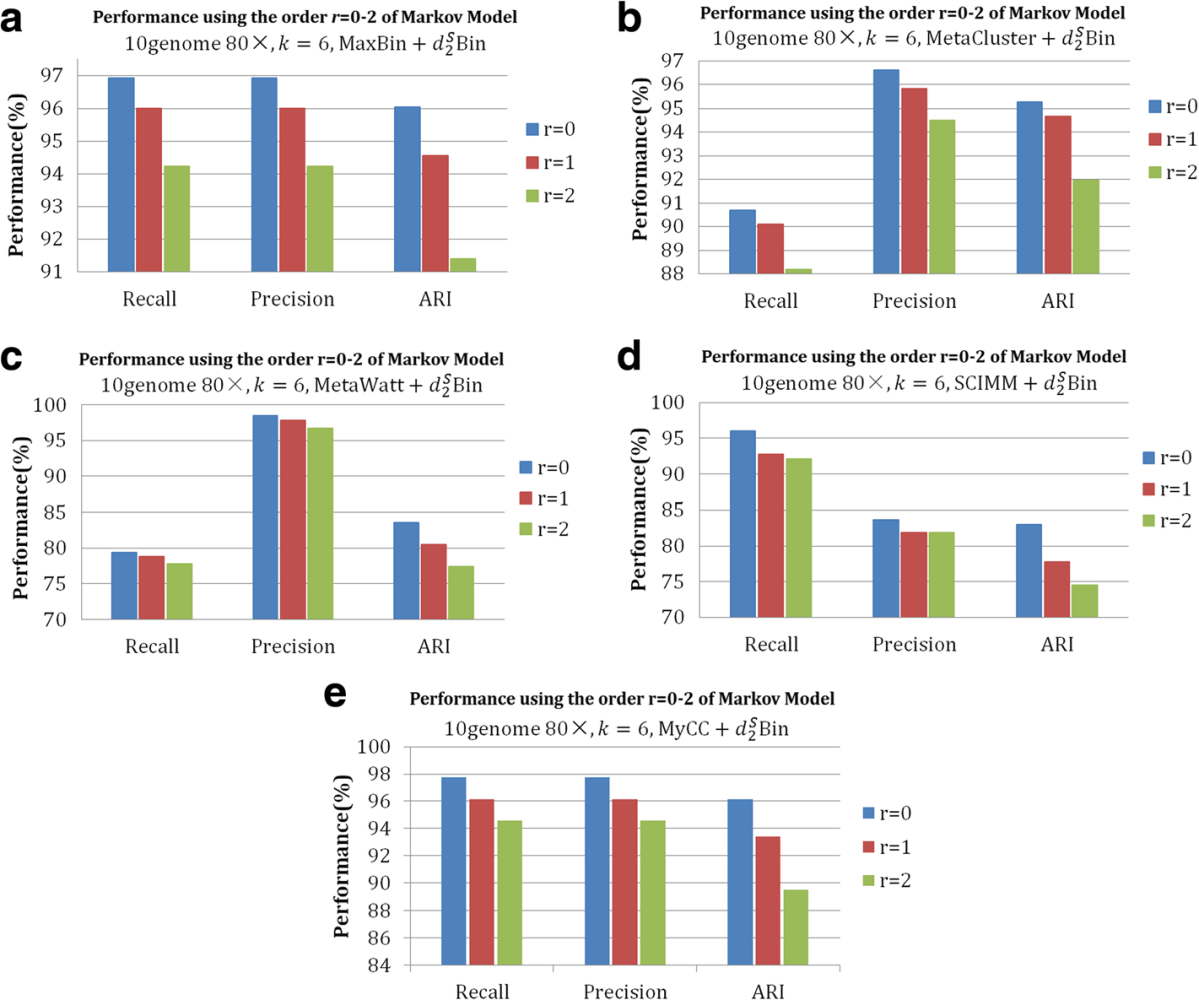
The effect of the order of Markov chain on the binning of contigs with different binning algorithms (MaxBin, MaxCluster, MetaWatt, SCIMM and MyCC) further modified by ${d}_2^S\mathrm{Bin}$ for 6-tuples on dataset *10genome 80×*. **a**-**e** are the *Recall*, *Precision* and *ARI* of 0–2 order of Markov chain to calculate ${d}_2^S\mathrm{Bin}$ on the five contig-binning tools. From the figures, it can be clearly seen that ${d}_2^S\mathrm{Bin}$ calculated on 0-order Markov chain achieves the best performance in all cases

**Fig. 5:**
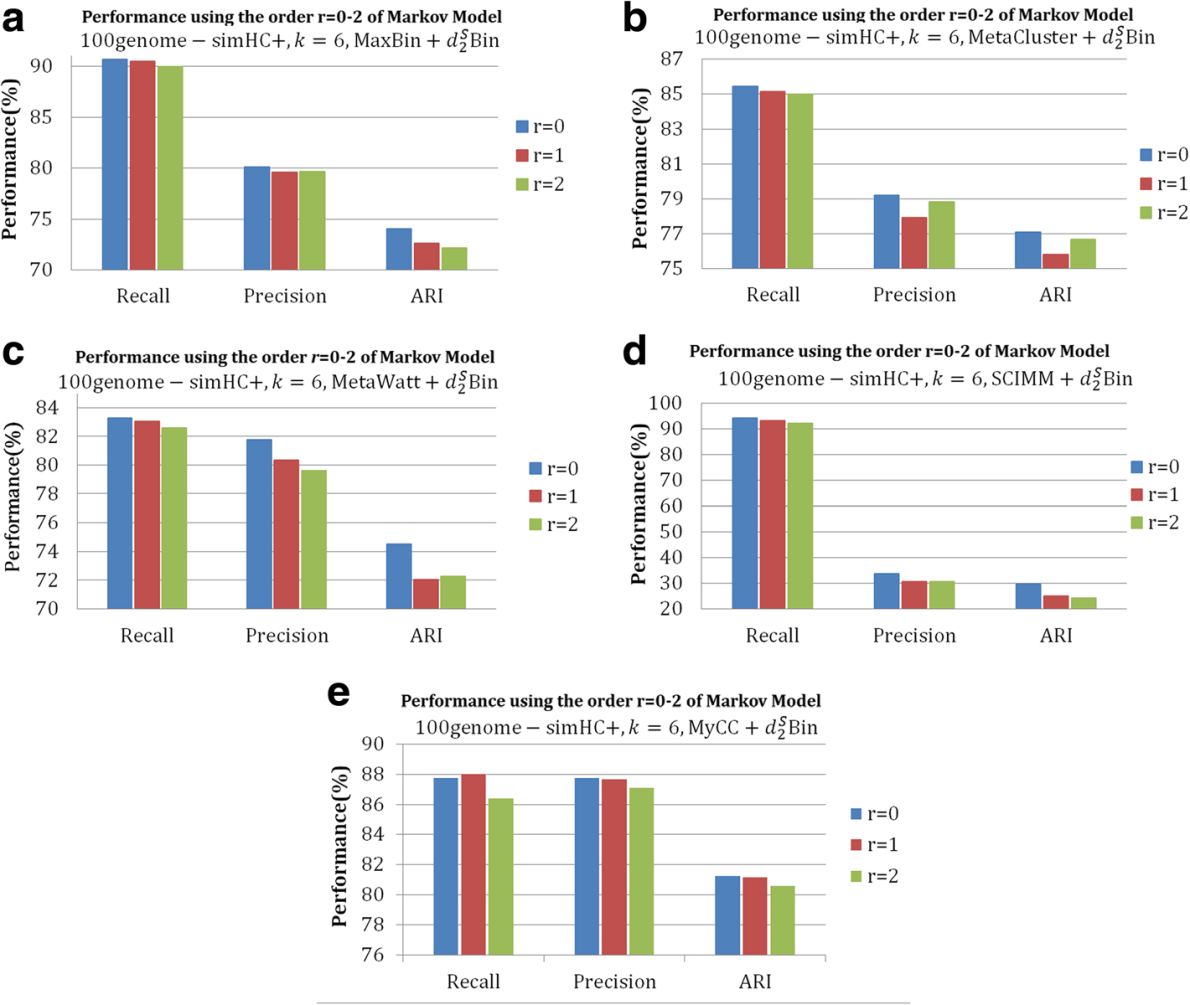
The effect of the order of Markov chain on the binning of contigs with different binning algorithms (MaxBin, MaxCluster, MetaWatt, SCIMM and MyCC) further modified by ${d}_2^S\mathrm{Bin}$ for 6-tuples on dataset *100genome simHC+*. **a**-**e** are the *Recall*, *Precision* and *ARI* of 0–2 order of Markov chain to calculate ${d}_2^S\mathrm{Bin}$ on the five contig-binning tools*.* From the figures, it can be clearly seen that ${d}_2^S\mathrm{Bin}$ calculated on 0-order Markov chain achieves the best performance in all cases

To obtain the most suitable Markov order for the background genome, we fixed the tuple length *k* = 6 and applied 0-2nd order Markov chain to calculate ${d}_2^S$ for datasets *10genome 80×* and *100genome-simHC+* on the output of five contig-binning tools. As shown in Figs. 4 and 5, for both datasets, ${d}_2^S\mathrm{Bin}$ under the i.i.d. model of 6-tuple achieves the best performance for *Precision*, *Recall* and *ARI* on all five tools. According to our previous studies about applying ${d}_2^S$ to compare metagenomic [28] and metatranscriptomic samples [26], ${d}_2^S$ under the i.i.d. model always achieved best results for all the 12 testing datasets, which illustrated that the i.i.d. model works well for the study of microbial communities. This is probably due to the fact that each bin is a mixture of several genomes and no Markov chain models with fixed order greater than 0 can describe the bin better. Therefore, we set tuple length *k* = 6 and the i.i.d. model in ${d}_2^S\mathrm{Bin}$.

### Experiments on contig binning

**Fig. 6:**
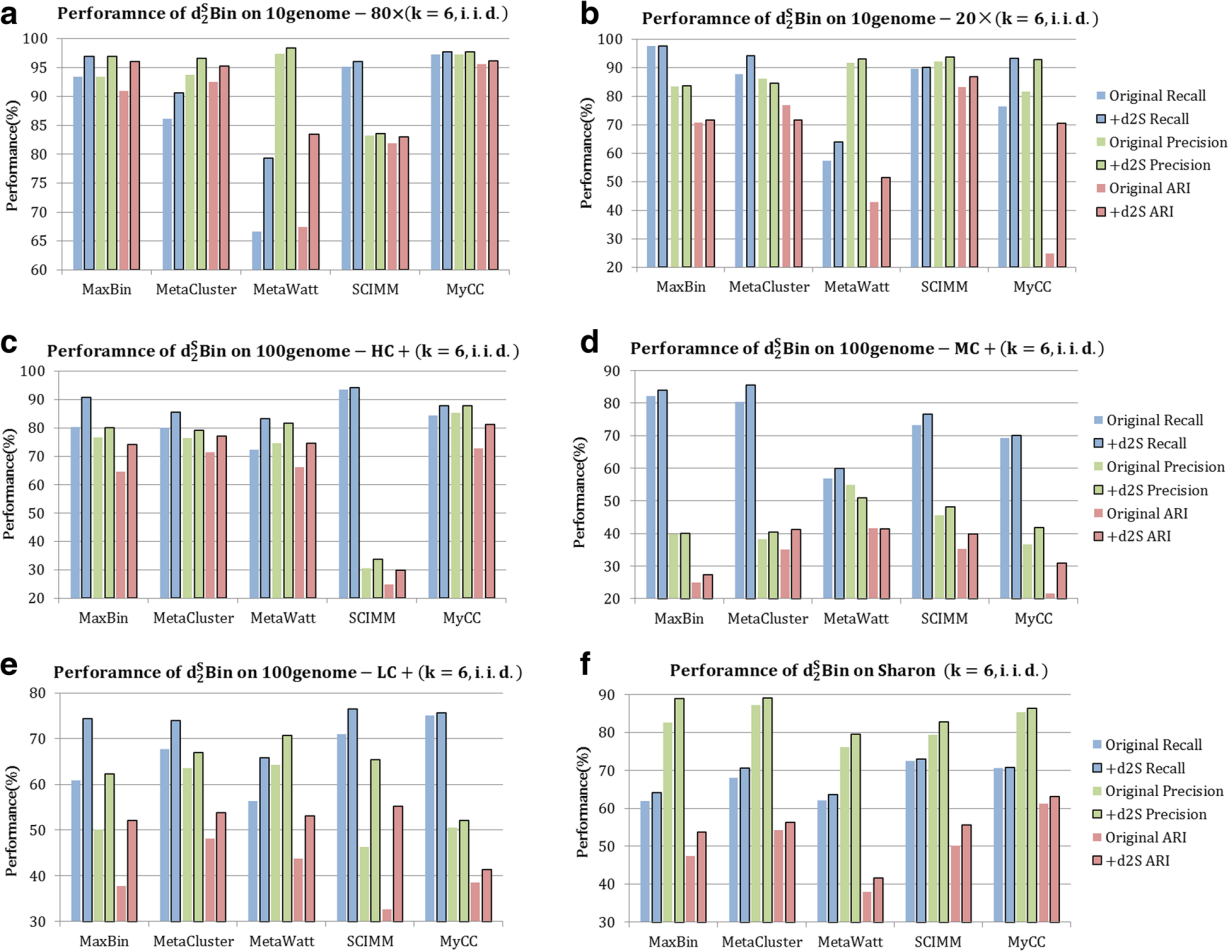
Contig binning on the six testing datasets. **a**-**f** are the results of six synthetic and real datasets for the five tools. The blue-, green- and red-colored bars are *recall*, *precision* and *ARI*, respectively. The bars without border are the criteria of the original outputs of the five tools. The bordered bars are the criteria after using ${d}_2^S\mathrm{Bin}$. It is obvious that performance increases in each case after adjustment by ${d}_2^S\mathrm{Bin}$

The contig-binning tools Maxbin [17], Metacluster 3.0 [14], Metawatt [3], SCIMM [11] and MyCC [20] were applied to bin the contigs from the six synthetic and real datasets with their original running modes. Based on the results from these tools, ${d}_2^S\mathrm{Bin}$ was further applied to adjust the contigs among bins. ${d}_2^S\mathrm{Bin}$ did not change the number of bins obtained by the original tools. The bar graphs in Fig. 6 illustrate the *Recall*, *Precision* and *ARI* of the output of the five existing tools and after the adjustment of ${d}_2^S\mathrm{Bin}$ for the six datasets. In most cases, the three criteria were improved by 1%–22%. Additional file 1: Table S1 presents the numerical values of the three indexes and offers more detailed information on all experiments, including the number of total&binned contigs and actual&clustered bins, providing more comprehensive view about the scale of dataset, complexity and original binning performance.

#### Contig binning on synthetic dataset *10 genome 80×* coverage

From Fig. 6a, it is easy to see that the three criteria were improved for all five tools. As shown in Additional file 1: Table S1, 8022 contigs were assembled from simulated metagenomic reads. The best results were obtained on MyCC where ${d}_2^S\mathrm{Bin}$ increased *recall*, *precision* and *ARI* from 97.21%, 97.21%, and 95.58% to 97.75%, 97.75% and 96.16%, respectively. MaxBin, MetaCluster and MyCC assigned the contigs into 10 bins. MetaWatt and SCIMM obtained 27 and 8 bins, respectively, but ${d}_2^S\mathrm{Bin}$ still adjusted contigs among these bins to achieve better performance.

#### Contig binning on synthetic dataset *10 genome 20×* coverage

Compared with 20 million reads in *10 genome 80×* data, *10 genome 20×* data have only 5 million reads for the 10 genomes. Fig. 6b shows that ${d}_2^{\mathrm{S}}\mathrm{Bin}$ improved the binning of MaxBin, MetaWatt, SCIMM and MyCC. As shown in Additional file 1: Table S1, both MaxBin and MetaCluster only produced three bins, and most contigs belonged to the three genomes with highest abundances because most contigs from the seven low-abundance genomes were discarded during preprocessing by having short length [17]. However, the ${d}_2^S\mathrm{Bin}$ only improved *precision*, but not *recall* or *ARI*, on MetaCluster. In order to have a deep insight on the deterioration of binning performance, we list the number of contigs from the 10 genomes in each bin, as shown in Additional file 1: Table S2–2 for MetaCluster and MetaCluster+ ${d}_2^S\mathrm{Bin}$. Each row of the table is one genome defined by its genome ID and corresponding genome name in NCBI and each column is the clustered bin, so the element is the number of contigs from one genome inside the current bin. Among the 1217 contigs assigned by MetaCluster, there are 1209 contigs from four dominant genomes: *Flavobacterium branchiophilum*, *Halothiobacillus neapolitanus*, *Lactobacillus casei* and *Acetobacter pasteurianus* with at least 100 contigs. But MetaCluster only output three bins: the contigs from *Flavobacterium branchiophilum*, *Halothiobacillus neapolitanus* and *Lactobacillus casei* are dominant in the three bins, and the contigs from *Acetobacter pasteurianus* are scattered into the three bins. After adjustment by ${d}_2^S\mathrm{Bin}$, the contigs from *Acetobacter pasteurianus* were merged into the same bin as *Halothiobacillus neapolitanus*. *Acetobacter pasteurianus* and *Halothiobacillus neapolitanus* are both from the phylum *Proteobacteria.* Therefore*, Acetobacter pasteurianus* is phylogenetically closer to *Halothiobacillus neapolitanus* than to the other two genomes*.* From this point of view, ${d}_2^S\mathrm{Bin}$ indeed improved the binning of MetaCluster although the performance index did not show improvement. Additional file 1: Table S2 also gives the details of contigs’ assignments in bins before and after ${d}_2^S\mathrm{Bin}$ for the other four tools. For MyCC in Additional file 1: TableS2–5, before using ${d}_2^S\mathrm{Bin}$, MyCC produced 5 bins and the contigs from *Halothiobacillus neapolitanus* were assigned to bin 1 and bin 4 and bin 1 included *Halothiobacillus neapolitanus* and *Lactobacillus casei,* which lead to the low *ARI* index as 24.76%. After using ${d}_2^S\mathrm{Bin}$, most contigs from *Halothiobacillus neapolitanus* were assigned to bin 4, and bin 1 mainly included contigs from *Lactobacillus casei*. The *ARI* was increased to 70.48%. The result demonstrates that ${d}_2^S\mathrm{Bin}$ tends to assign contigs with consistent or similar background models to the same bin.

#### Contig binning on synthetic dataset *100 genome-simHC+*

*simHC+* has evenly distributed species abundance levels with no dominant species. According to Fig. 6c, the three criteria were all improved for the five tools. According to Additional file 1: Table S1, among a total of 407,873 contigs, 13,919 were clustered into 87 bins by MaxBin with 80.23%, 76.69 and 64.58% *recall*, *precision* and *ARI,* respectively. After ${d}_2^S\mathrm{Bin}$, the three indexes were improved to 90.67%, 80.14% and 74.03%, respectively, showing overall superior performance. MetaCluster, MetaWatt, and MyCC produced 97, 129 and 94 bins, respectively, and *recall*, *precision* and *ARI* were improved for all of them by ${d}_2^S\mathrm{Bin}$. SCIMM only clustered 19 bins, which led to low *precision* and *ARI*, but ${d}_2^S\mathrm{Bin}$ still improved the three metrics.

#### Contig binning on synthetic dataset *100 genome-simMC+*

According to Fig. 6d, the three criteria were improved by ${d}_2^S\mathrm{Bin}$ for MaxBin, MetaCluster, SCIMM and MyCC. Owing to the poor assembly quality of *simMC+* [17], only ~10,000+ contigs of the 795,573 passed the minimum length threshold, among which a small portion came from low-abundance genomes. Therefore, only high-abundance genomes were binned, and 11 bins were generated for MaxBin and MetaCluster, and 15 bins for MyCC. The large disparity between the number of real species and bins led to low *precision* and *ARI*. However, ${d}_2^S\mathrm{Bin}$ still greatly improved *recall*, *precision* and *ARI*. The exception was MetaWatt. Among the 11,987 clustered contigs, MetaWatt isolated 41 bins. In this case, extracting contigs from the dominant genome from each bin would leave only 7978, meaning that one-third of the contigs would remain to interfere with the modeling of the 41 dominant genomes, in turn leading to decreased performance for *precision* and *ARI*.

#### Contig binning on synthetic dataset *100 genome-simLC+*

${d}_2^S\mathrm{Bin}$ improved the binning performance for all tools. All three metrics were also significantly improved by ${d}_2^S\mathrm{Bin}$. For SCIMM, ${d}_2^S\mathrm{Bin}$ increased *recall*, *precision* and *ARI* from 70.99%, 46.29% and 32.64% to 76.42%, 65.46% and 55.24%, respectively, which represents the best performance among the five tools.

#### Contig binning on real dataset *Sharon*

**Fig. 7:**
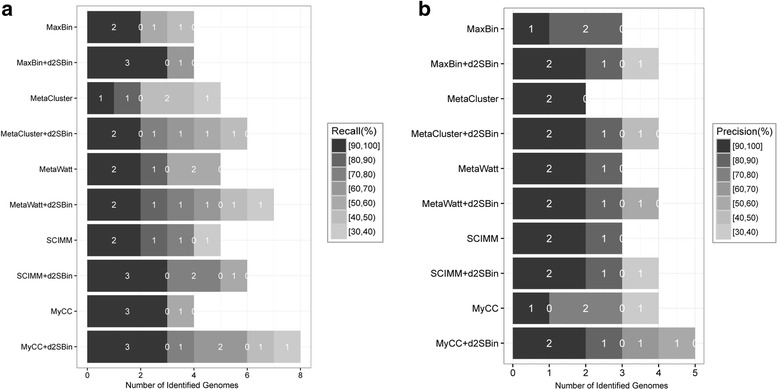
Evaluation of *recall* and *precision* of the *Sharon* dataset with CheckM. **a** The plot shows the number of recovered genome bins (X-axis) by each method (Y-axis) at different *recall* (completeness) thresholds (gray scale) with *precision* (lack of contamination) ≥ 80%. **b** The plot shows the number of recovered genome bins (X-axis) by each method (Y-axis) at different *precision* thresholds (gray scale) with *recall* ≥ 80%. It is clear that ${d}_2^S\mathrm{Bin}$ improved the *recall* and *precision* of each bin compared with the original tools. The number “0” shown on the border means that one or more value intervals were skipped because no genome was recovered in the intervals

For this real dataset, the ground truth of binning was not available. The following two evaluations were implemented: (1) We only binned the 2614 contigs with unambiguous labels belonging to 21 species, and the annotations were considered as the ground truth. MaxBin, MetaCluster, MetaWatt, SCIMM and MyCC isolated 11, 10, 23, 19 and 16 bins for *Sharon* originally. As shown in Fig. 6f, based on their binning outputs, ${d}_2^S\mathrm{Bin}$ adjusted the contig binning and increased *Recall*, *Precision* and *ARI* for all tools. (2) We applied CheckM [40] to estimate the approximate contamination and genome completeness of the contigs in the bins free from ground truth. Figure 7a shows the number of recovered genome bins by each method in different *recall* (completeness) threshold with *precision* (lack of contamination) > 80%. Although the tools identified 10–23 bins among the 21 species in the Sharon dataset, only 4–6 genome bins were recovered with *precision* > 80%. ${d}_2^S\mathrm{Bin}$ did improve *recall* and *precision*. For MetaWatt and MyCC, ${d}_2^S\mathrm{Bin}$ increased the number of bins with *precision* > 80%. For MetaCluster and SCIMM, ${d}_2^S\mathrm{Bin}$ not only increased the number of bins with *precision* > 80% but also increased the number of bins with *recall* > 90%. The ${d}_2^S\mathrm{Bin}$ also increased the *recall* of each bin for MaxBin and MyCC. Figure 7b shows the number of recovered genome bins at different *precision* thresholds with *recall* > 80%. For all tools, ${d}_2^S\mathrm{Bin}$ increased the number of bins with *recall* > 80%. For MaxBin and MyCC, the number of bins with *precision* > 90% is also increased by ${d}_2^S\mathrm{Bin}$.

Testing on these synthetic and real datasets showed that ${d}_2^S\mathrm{Bin}$ could achieve obvious improvement on the original outputs of the five testing tools.

#### Convergence of K-means iteration on ${d}_2^S\mathrm{Bin}$

**Fig. 8:**
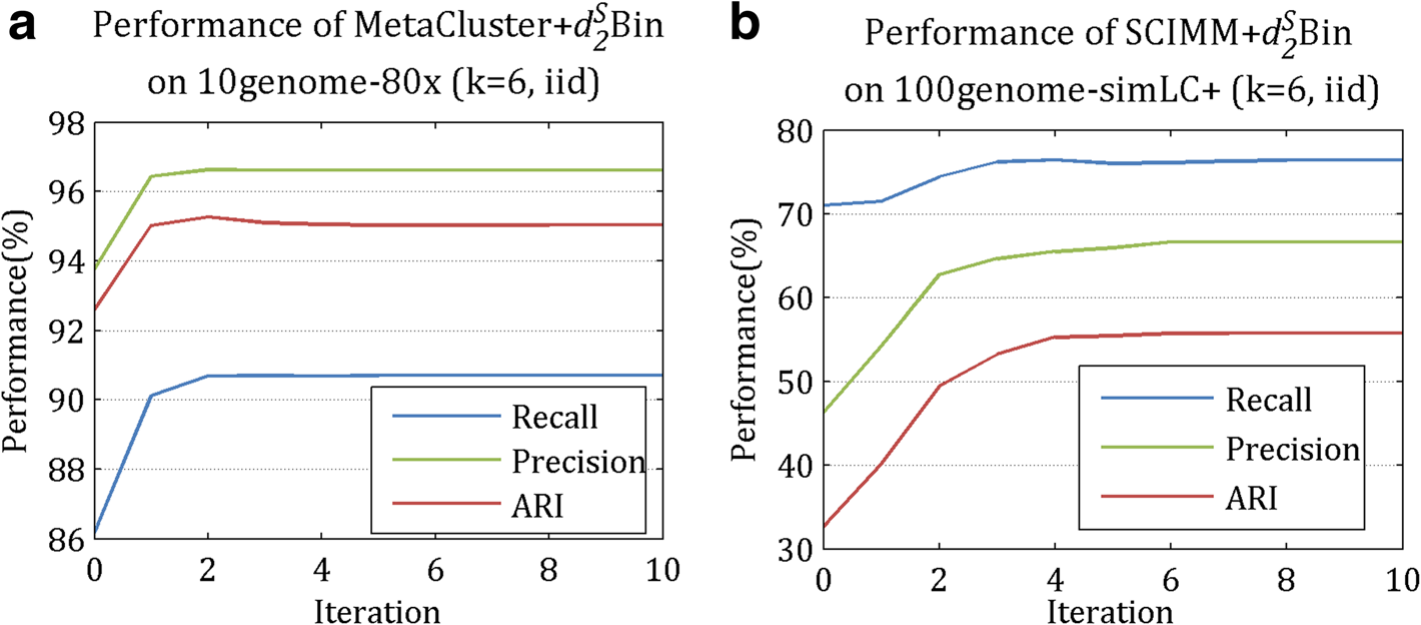
Curves of the three indexes with the K-means iterations. The “0” in the horizontal ordinate reflects the output performance of the original binning tool, MetaCluster in (**a**) and SCIMM in (**b**). The three indexes increase significantly on the first iteration, followed by slight adjustment to reach steady values

In order to evaluate the convergence of K-means iteration on ${d}_2^S\mathrm{Bin}$, we plotted the performance curves of the three indexes on randomly selected tools and datasets, as shown in Fig. 8. During our experiments with ten iterations, the three indexes increased significantly on the first iteration and reached steady state quickly. The “0” in the horizontal ordinate indicates the performance of the original binning tool. Therefore, in ${d}_2^S\mathrm{Bin}$, the iterations of contig binning with K-means will stop when no contigs is adjusted or the number of iterations reaches 5.

### Software implementation and running

The code of ${d}_2^S\mathrm{Bin}$ was implemented with Python and Cython running under the Linux system. Cython is a superset of the Python language that additionally supports calling C functions, and the code can be compiled into a sharing library called by python directly. Tested on a server with 128G memory and Intel(R) Xeon(R) CPU E5–2620 v2 @ 2.10GHz with 6 CPU cores at 2.10 GHz, it takes 16 min to finish the adjustment of contig binning for ${d}_2^S\mathrm{Bin}$ on 6-tuples for 8022 contigs of 10 bins with 4000 bp length on average and the peak memory is 6.7GB. The source code of ${d}_2^S\mathrm{Bin}$ is available at https://github.com/kunWangkun/d2SBin.

## Discussion

Our experiments demonstrate ${d}_2^S$ can measure the similarity between contigs more accurately. However, ${d}_2^S$ requires to build the background Markov model for each contig, which bring heavy computation burden. Therefore, in our study, instead of de novo binning from scratch, we attempt to adjust contig bins based on the output of any existing binning tools for the single metagenomic sample. The computational issue can be overcome using this strategy. When there are multiple related samples available, the sequence composition contribute less than the co-varying coverage profiles across samples for contig binning and ${d}_2^S\mathrm{Bin}$ can not improve the contig binning for multiple metagenomic samples. The tools designed for multiple samples, like COCACOLA, GroopM, Concoct, MaxBin2.0, can achieve satisfactory results if multiple metagenomic samples are available.

Currently, ${d}_2^S\mathrm{Bin}$ does not merge, or split, the bins. In some situations that there may be large differences between the numbers of clustered bins and ground truth, merging and splitting the bins would improve the results. However, the algorithms to adjust the clustering number, such as ISODATA [41], require the inputs of the minimum threshold of between-class dissimilarity and the maximum threshold of within-class dissimilarity. These thresholds depend on the detailed taxonomic level which the investigators are interested in. Once these thresholds are given, we can combine the algorithms for merging and splitting bins with ${d}_2^S\mathrm{Bin}$ to further improve the binning results.

## Conclusions

The ability of ${d}_2^S\mathrm{Bin}$ to achieve improved binning performance is based on the idea that contigs clustered into one bin will come from the same genome and that relative sequence compositions will be similar across different regions of the same genome, but differ between genomes [21, 22]. ${d}_2^S$ measures the dissimilarity between contig and the bin’s center based on the Markov model of *k*-tuple sequence compositions.

Our experiments demonstrate that ${d}_2^S\mathrm{Bin}$ significantly improves binning performance in almost all cases, thus giving credence to the relative sequence composition model over the direct application of absolute sequence composition. We applied ${d}_2^S\mathrm{Bin}$ to five contig-binning tools with different binning strategies. Irrespective of the different strategies employed by the contig-binning tools, ${d}_2^S\mathrm{Bin}$ was able to achieve better performance for all tools tested. Finally, the optimal results for ${d}_2^S\mathrm{Bin}$ are always obtained on steady tuple length *k* = 6 under the i.i.d. model with no need to search for the optimal parameters.

## Additional file

Additional file 1: Table S1. The file gives the numerical values of three criteria of contig binning on the experiments of the six testing datasets. **Table S2.** Detailed binning results of the contigs before and after ${d}_2^S\mathrm{Bin}$ for dataset *10genome-20×* based on the five testing tools. (DOCX 38 kb)

## Abbreviations

*ARI*: Adjusted rand index
EM: Expectation maximization
*i.i.d.*: independent identically distributed
