# Supplementary material for: Improving contig binning of metagenomic data using d2S oligonucleotide frequency dissimilarity
Source: BMC Bioinformatics. 2017 Sep 20;18:425. doi: 10.1186/s12859-017-1835-1 (PMC5607646; doi:10.1186/s12859-017-1835-1)
Supplement: Additional file 1: Table S1. — The file gives the numerical values of three criteria of contig binning on the experiments of the six testing datasets. Table S2. Detailed binning results of the contigs before and after ${d}_2^S\mathrm{Bin}$d2SBin for dataset 10genome-20× based on the five testing tools. (DOCX 38 kb) [file 12859_2017_1835_MOESM1_ESM.docx]

**Table S1. Contigs binning on six testing datasets: the performance of** $\boldsymbol{d}_{\boldsymbol{2}}^{\boldsymbol{s}}\mathbf{Bin}$ **(k=6, 0-order Markov Chain) on the output of MaxBin1.0, MetaCluster 3.0, MetaWatt and SCIMM**

| **Datasets** | **Tools** | **#Total Contigs** | **#Binned Contigs** | **#Bin** | ***Recall*%** | ***Precision*%** | ***ARI*%** |  |
| --- | --- | --- | --- | --- | --- | --- | --- | --- |
| ***10genome-80×*** | **MaxBin1.0** | 15701  (10 species) | 8022 | 10 | 93.48 | 93.48 | 90.96 |  |
|  | $\boldsymbol{+d}_{\boldsymbol{2}}^{\boldsymbol{s}}\mathbf{Bin}$ |  |  |  | 96.93 | 96.93 | 96.05 |  |
|  | **MetaCluster3.0** |  | 7721 | 10 | 86.18 | 93.76 | 92.58 | |
|  | $\boldsymbol{+d}_{\boldsymbol{2}}^{\boldsymbol{s}}\mathbf{Bin}$ |  |  |  | 90.69 | 96.63 | 95.26 | |
|  | **MetaWatt** |  | 7701 | 27 | 66.62 | 97.33 | 67.42 | |
|  | $\boldsymbol{+d}_{\boldsymbol{2}}^{\boldsymbol{s}}\mathbf{Bin}$ |  |  |  | 79.32 | 98.43 | 83.52 | |
|  | **SCIMM** |  | 8802 | 8 | 95.10 | 83.22 | 81.88 | |
|  | $\boldsymbol{+d}_{\boldsymbol{2}}^{\boldsymbol{s}}\mathbf{Bin}$ |  |  |  | 95.99 | 83.62 | 82.98 | |
|  | **MyCC** |  | 7721 | 10 | 97.21 | 97.21 | 95.58 | |
|  | $\boldsymbol{+d}_{\boldsymbol{2}}^{\boldsymbol{s}}\mathbf{Bin}$ |  |  |  | 97.75 | 97.75 | 96.16 | |
| ***10genome-20×*** | **MaxBin1.0** | 40812  (10 species) | 1260 | 3 | 97.62 | 83.41 | 70.76 |  |
|  | $\boldsymbol{+d}_{\boldsymbol{2}}^{\boldsymbol{s}}\mathbf{Bin}$ |  |  |  | 97.62 | 83.73 | 71.62 |  |
|  | **MetaCluster3.0** |  | 1217 | 3 | 87.78 | 86.20 | 76.85 | |
|  | $\boldsymbol{+d}_{\boldsymbol{2}}^{\boldsymbol{s}}\mathbf{Bin}$ |  |  |  | 94.21 | 84.47 | 71.57 | |
|  | **MetaWatt** |  | 1147 | 12 | 57.19 | 91.54 | 42.73 | |
|  | $\boldsymbol{+d}_{\boldsymbol{2}}^{\boldsymbol{s}}\mathbf{Bin}$ |  |  |  | 63.99 | 93.02 | 51.35 | |
|  | **SCIMM** |  | 1260 | 8 | 89.60 | 92.14 | 83.25 | |
|  | $\boldsymbol{+d}_{\boldsymbol{2}}^{\boldsymbol{s}}\mathbf{Bin}$ |  |  |  | 89.92 | 93.73 | 86.76 | |
|  | **MyCC** |  | 1260 | 5 | 76.42 | 81.58 | 24.76 | |
|  | $\boldsymbol{+d}_{\boldsymbol{2}}^{\boldsymbol{s}}\mathbf{Bin}$ |  |  |  | 93.10 | 92.78 | 70.48 | |
| ***100 genome- simHC+*** | **MaxBin1.0** | 407873  (100 species) | 13919 | 87 | 80.23 | 76.69 | 64.58 |  |
|  | $\boldsymbol{+d}_{\boldsymbol{2}}^{\boldsymbol{s}}\mathbf{Bin}$ |  |  |  | 90.67 | 80.14 | 74.03 |  |
|  | **MetaCluster3.0** |  | 13899 | 97 | 80.01 | 76.36 | 71.39 | |
|  | $\boldsymbol{+d}_{\boldsymbol{2}}^{\boldsymbol{s}}\mathbf{Bin}$ |  |  |  | 85.42 | 79.21 | 77.08 | |
|  | **MetaWatt** |  | 13799 | 129 | 72.29 | 74.59 | 66.08 | |
|  | $\boldsymbol{+d}_{\boldsymbol{2}}^{\boldsymbol{s}}\mathbf{Bin}$ |  |  |  | 83.28 | 81.75 | 74.49 | |
|  | **SCIMM** |  | 13919 | 19 | 93.52 | 30.49 | 24.93 | |
|  | $\boldsymbol{+d}_{\boldsymbol{2}}^{\boldsymbol{s}}\mathbf{Bin}$ |  |  |  | 94.09 | 33.84 | 29.78 | |
|  | **MyCC** |  | 13919 | 94 | 84.41 | 85.23 | 72.86 | |
|  | $\boldsymbol{+d}_{\boldsymbol{2}}^{\boldsymbol{s}}\mathbf{Bin}$ |  |  |  | 87.77 | 87.74 | 81.25 | |
| ***100 genome- simMC+*** | **MaxBin1.0** | 795573  (100 species) | 13012 | 11 | 82.15 | 39.72 | 24.85 |  |
|  | $\boldsymbol{+d}_{\boldsymbol{2}}^{\boldsymbol{s}}\mathbf{Bin}$ |  |  |  | 83.98 | 39.96 | 27.32 |  |
|  | **MetaCluster3.0** |  | 12497 | 11 | 80.37 | 38.16 | 35.10 | |
|  | $\boldsymbol{+d}_{\boldsymbol{2}}^{\boldsymbol{s}}\mathbf{Bin}$ |  |  |  | 85.40 | 40.45 | 41.13 | |
|  | **MetaWatt** |  | 11987 | 41 | 56.86 | 54.92 | 41.59 | |
|  | $\boldsymbol{+d}_{\boldsymbol{2}}^{\boldsymbol{s}}\mathbf{Bin}$ |  |  |  | 59.86 | 50.92 | 41.39 | |
|  | **SCIMM** |  | 13012 | 86 | 73.15 | 45.67 | 35.29 | |
|  | $\boldsymbol{+d}_{\boldsymbol{2}}^{\boldsymbol{s}}\mathbf{Bin}$ |  |  |  | 76.51 | 48.20 | 39.74 | |
|  | **MyCC** |  | 13012 | 15 | 69.18 | 36.68 | 21.52 | |
|  | $\boldsymbol{+d}_{\boldsymbol{2}}^{\boldsymbol{s}}\mathbf{Bin}$ |  |  |  | 69.98 | 41.85 | 30.94 | |
| ***100 genome- simLC+*** | **MaxBin1.0** | 407873  (100 species) | 84698 | 77 | 60.85 | 50.16 | 37.77 |  |
|  | $\boldsymbol{+d}_{\boldsymbol{2}}^{\boldsymbol{s}}\mathbf{Bin}$ |  |  |  | 74.33 | 62.34 | 52.11 |  |
|  | **MetaCluster3.0** |  | 81535 | 75 | 67.71 | 63.58 | 48.07 | |
|  | $\boldsymbol{+d}_{\boldsymbol{2}}^{\boldsymbol{s}}\mathbf{Bin}$ |  |  |  | 73.91 | 66.98 | 53.74 | |
|  | **MetaWatt** |  | 83566 | 145 | 56.39 | 64.30 | 43.67 | |
|  | $\boldsymbol{+d}_{\boldsymbol{2}}^{\boldsymbol{s}}\mathbf{Bin}$ |  |  |  | 65.78 | 70.67 | 53.04 | |
|  | **SCIMM** |  | 84698 | 61 | 70.99 | 46.29 | 32.64 | |
|  | $\boldsymbol{+d}_{\boldsymbol{2}}^{\boldsymbol{s}}\mathbf{Bin}$ |  |  |  | 76.42 | 65.46 | 55.24 | |
|  | **MyCC** |  | 84698 | 54 | 75.10 | 50.50 | 38.49 | |
|  | $\boldsymbol{+d}_{\boldsymbol{2}}^{\boldsymbol{s}}\mathbf{Bin}$ |  |  |  | 75.68 | 52.06 | 41.37 | |
| ***Sharon*** | **MaxBin1.0** | 5579  (21 species) | 1975 | 11 | 61.87 | 82.63 | 47.39 |  |
|  | $\boldsymbol{+d}_{\boldsymbol{2}}^{\boldsymbol{s}}\mathbf{Bin}$ |  |  |  | 64.15 | 88.86 | 53.70 |  |
|  | **MetaCluster3.0** |  | 1905 | 10 | 68.13 | 87.13 | 54.15 | |
|  | $\boldsymbol{+d}_{\boldsymbol{2}}^{\boldsymbol{s}}\mathbf{Bin}$ |  |  |  | 70.55 | 89.08 | 56.20 | |
|  | **MetaWatt** |  | 2455 | 23 | 62.12 | 76.13 | 37.91 | |
|  | $\boldsymbol{+d}_{\boldsymbol{2}}^{\boldsymbol{s}}\mathbf{Bin}$ |  |  |  | 63.54 | 79.43 | 41.55 | |
|  | **SCIMM** |  | 2614 | 19 | 72.57 | 79.29 | 50.18 | |
|  | $\boldsymbol{+d}_{\boldsymbol{2}}^{\boldsymbol{s}}\mathbf{Bin}$ |  |  |  | 73.08 | 82.67 | 55.53 | |
|  | **MyCC** |  | 1905 | 16 | 70.58 | 85.26 | 61.17 | |
|  | $\boldsymbol{+d}_{\boldsymbol{2}}^{\boldsymbol{s}}\mathbf{Bin}$ |  |  |  | 70.78 | 86.38 | 63.02 | |

“#Total contigs” means the number of contigs assembled from the high throughout sequencing reads. “#Binned Contigs” means the number of contigs pass the filtering and binned by the tool. “#Bin” means the number of produced bins by the current tool.

**Table S2.** **Detailed binning results of the contigs before and after** $\boldsymbol{d}_{\boldsymbol{2}}^{\boldsymbol{s}}\mathbf{Bin}$**, ground truth for dataset *10genome-20×* based on the five testing tools**

**TableS2-1**

| **Tool** | **NCBI Genome ID** | **Genome Name** | **Bin** | | | **Number of contigs belong to the genome** |
| --- | --- | --- | --- | --- | --- | --- |
|  |  |  | **1** | **2** | **3** |  |
| **MaxBin1.0** | 256653503 | *Acetobacter pasteurianus IFO 3283-12 DNA* | 1 | 4 | 97 | 102 |
|  | 347534971 | *Flavobacterium branchiophilum FL-15* | 0 | 434 | 10 | 444 |
|  | 261854630 | *Halothiobacillus neapolitanus c2* | 520 | 2 | 3 | 525 |
|  | 300112745 | *Nitrosococcus watsoni C-113* | 1 | 0 | 0 | 1 |
|  | 327384027 | *Lactobacillus casei BD-II* | 166 | 1 | 5 | 172 |
|  | 325278757 | *Odoribacter splanchnicus DSM 20712* | 4 | 1 | 0 | 5 |
|  | 111017022 | *Rhodococcus jostii RHA1* | 2 | 1 | 0 | 3 |
|  | 182682970 | *Streptococcus pneumoniae CGSP14* | 3 | 1 | 0 | 4 |
|  | 307723218 | *Thermoanaerobacter sp. X513* | 1 | 1 | 0 | 2 |
|  | 154243958 | *Xanthobacter autotrophicus Py2* | 2 | 0 | 0 | 2 |
| **MaxBin1.0**  $\boldsymbol{+d}_{\boldsymbol{2}}^{\boldsymbol{s}}\mathbf{Bin}$ | 256653503 | *Acetobacter pasteurianus IFO 3283-12 DNA* | 10 | 6 | 86 | 102 |
|  | 347534971 | *Flavobacterium branchiophilum FL-15* | 0 | 444 | 0 | 444 |
|  | 261854630 | *Halothiobacillus neapolitanus c2* | 525 | 0 | 0 | 525 |
|  | 300112745 | *Nitrosococcus watsoni C-113* | 0 | 1 | 0 | 1 |
|  | 327384027 | *Lactobacillus casei BD-II* | 161 | 8 | 3 | 172 |
|  | 325278757 | *Odoribacter splanchnicus DSM 20712* | 0 | 4 | 1 | 5 |
|  | 111017022 | *Rhodococcus jostii RHA1* | 1 | 2 | 0 | 3 |
|  | 182682970 | *Streptococcus pneumoniae CGSP14* | 1 | 3 | 0 | 4 |
|  | 307723218 | *Thermoanaerobacter sp. X513* | 0 | 2 | 0 | 2 |
|  | 154243958 | *Xanthobacter autotrophicus Py2* | 2 | 0 | 0 | 2 |

**TableS2-2**

| **Tool** | **NCBI Genome ID** | **Genome Name** | **Bin** | | | **Number of contigs belong to the genome** |
| --- | --- | --- | --- | --- | --- | --- |
|  |  |  | **1** | **2** | **3** |  |
| **MetaCluster3** | 347534971 | *Flavobacterium branchiophilum FL-15* | 0 | 0 | 436 | 436 |
|  | 261854630 | *Halothiobacillus neapolitanus c2* | 473 | 41 | 4 | 518 |
|  | 327384027 | *Lactobacillus casei BD-II* | 6 | 140 | 13 | 159 |
|  | 256653503 | *Acetobacter pasteurianus IFO 3283-12 DNA* | 36 | 31 | 29 | 96 |
|  | 300112745 | *Nitrosococcus watsoni C-113* | 0 | 0 | 0 | 0 |
|  | 325278757 | *Odoribacter splanchnicus DSM 20712* | 1 | 0 | 1 | 2 |
|  | 111017022 | *Rhodococcus jostii RHA1* | 1 | 0 | 0 | 1 |
|  | 182682970 | *Streptococcus pneumoniae CGSP14* | 0 | 0 | 1 | 1 |
|  | 307723218 | *Thermoanaerobacter sp. X513* | 0 | 1 | 1 | 2 |
|  | 154243958 | *Xanthobacter autotrophicus Py2* | 1 | 1 | 0 | 2 |
| **MetaCluster3**  $\boldsymbol{+d}_{\boldsymbol{2}}^{\boldsymbol{s}}\mathbf{Bin}$ | 347534971 | *Flavobacterium branchiophilum FL-15* | 9 | 3 | 424 | 436 |
|  | 261854630 | *Halothiobacillus neapolitanus c2* | 472 | 42 | 4 | 518 |
|  | 327384027 | *Lactobacillus casei BD-II* | 8 | 132 | 19 | 159 |
|  | 256653503 | *Acetobacter pasteurianus IFO 3283-12 DNA* | 96 | 0 | 0 | 96 |
|  | 300112745 | *Nitrosococcus watsoni C-113* | 0 | 0 | 0 | 0 |
|  | 325278757 | *Odoribacter splanchnicus DSM 20712* | 0 | 2 | 0 | 2 |
|  | 111017022 | *Rhodococcus jostii RHA1* | 1 | 0 | 0 | 1 |
|  | 182682970 | *Streptococcus pneumoniae CGSP14* | 0 | 0 | 1 | 1 |
|  | 307723218 | *Thermoanaerobacter sp. X513* | 0 | 0 | 2 | 2 |
|  | 154243958 | *Xanthobacter autotrophicus Py2* | 2 | 0 | 0 | 2 |

**TableS2-3**

| **Tool** | **NCBI**  **Genome ID** | **Genome Name** | **Bin** | | | | | | | | | | | | **Number**  **of contigs belong to the genome** |
| --- | --- | --- | --- | --- | --- | --- | --- | --- | --- | --- | --- | --- | --- | --- | --- |
|  |  |  | **1** | **2** | **3** | **4** | **5** | 6 | **7** | **8** | **9** | **10** | **11** | **12** |  |
| **MetaWatt** | 256653503 | *Acetobacter pasteurianus IFO 3283-12 DNA* | 0 | 2 | 0 | 4 | 5 | 9 | 30 | 0 | 0 | 0 | 0 | 9 | 59 |
|  | 347534971 | *Flavobacterium branchiophilum FL-15* | 48 | 0 | 0 | 0 | 0 | 0 | 0 | 178 | 121 | 0 | 45 | 0 | 392 |
|  | 261854630 | *Halothiobacillus neapolitanus c2* | 0 | 47 | 73 | 7 | 0 | 0 | 0 | 0 | 0 | 339 | 2 | 52 | 520 |
|  | 300112745 | *Nitrosococcus watsoni C-113* | 0 | 0 | 0 | 0 | 0 | 0 | 0 | 0 | 0 | 0 | 0 | 0 | 0 |
|  | 327384027 | *Lactobacillus casei BD-II* | 0 | 0 | 18 | 103 | 0 | 0 | 0 | 0 | 0 | 1 | 4 | 42 | 168 |
|  | 325278757 | *Odoribacter splanchnicus DSM 20712* | 0 | 0 | 0 | 2 | 0 | 0 | 0 | 0 | 0 | 0 | 1 | 1 | 4 |
|  | 111017022 | *Rhodococcus jostii RHA1* | 0 | 0 | 0 | 0 | 0 | 0 | 0 | 0 | 0 | 0 | 0 | 0 | 0 |
|  | 182682970 | *Streptococcus pneumoniae CGSP14* | 0 | 0 | 0 | 2 | 0 | 0 | 0 | 0 | 0 | 0 | 0 | 0 | 2 |
|  | 307723218 | *Thermoanaerobacter sp. X513* | 0 | 0 | 0 | 0 | 0 | 0 | 0 | 0 | 0 | 0 | 0 | 0 | 0 |
|  | 154243958 | *Xanthobacter autotrophicus Py2* | 0 | 2 | 0 | 0 | 0 | 0 | 0 | 0 | 0 | 0 | 0 | 0 | 2 |
| **MetaWatt**  $\boldsymbol{+d}_{\boldsymbol{2}}^{\boldsymbol{s}}\mathbf{Bin}$ | 256653503 | *Acetobacter pasteurianus IFO 3283-12 DNA* | 1 | 2 | 0 | 2 | 9 | 9 | 32 | 0 | 0 | 1 | 0 | 3 | 59 |
|  | 347534971 | *Flavobacterium branchiophilum FL-15* | 75 | 0 | 0 | 0 | 0 | 0 | 0 | 205 | 52 | 0 | 60 | 0 | 392 |
|  | 261854630 | *Halothiobacillus neapolitanus c2* | 0 | 50 | 55 | 4 | 0 | 0 | 0 | 0 | 1 | 382 | 1 | 27 | 520 |
|  | 300112745 | *Nitrosococcus watsoni C-113* | 0 | 0 | 0 | 0 | 0 | 0 | 0 | 0 | 0 | 0 | 0 | 0 | 0 |
|  | 327384027 | *Lactobacillus casei BD-II* | 1 | 1 | 24 | 108 | 0 | 0 | 0 | 0 | 1 | 2 | 1 | 30 | 168 |
|  | 325278757 | *Odoribacter splanchnicus DSM 20712* | 1 | 0 | 0 | 1 | 0 | 0 | 0 | 0 | 0 | 0 | 1 | 1 | 4 |
|  | 111017022 | *Rhodococcus jostii RHA1* | 0 | 0 | 0 | 0 | 0 | 0 | 0 | 0 | 0 | 0 | 0 | 0 | 0 |
|  | 182682970 | *Streptococcus pneumoniae CGSP14* | 0 | 0 | 0 | 1 | 0 | 0 | 0 | 0 | 0 | 0 | 1 | 0 | 2 |
|  | 307723218 | *Thermoanaerobacter sp. X513* | 0 | 0 | 0 | 0 | 0 | 0 | 0 | 0 | 0 | 0 | 0 | 0 | 0 |
|  | 154243958 | *Xanthobacter autotrophicus Py2* | 0 | 2 | 0 | 0 | 0 | 0 | 0 | 0 | 0 | 0 | 0 | 0 | 2 |

**TableS2-4**

| **Tool** | **NCBI Genome ID** | **Genome Name** | **Bin** | | | | | | | | **Number of contigs belong to the genome** |
| --- | --- | --- | --- | --- | --- | --- | --- | --- | --- | --- | --- |
|  |  |  | **1** | **2** | **3** | **4** | **5** | 6 | **7** | **8** |  |
| **SCIMM** | 256653503 | *Acetobacter pasteurianus IFO 3283-12 DNA* | 8 | 0 | 0 | 0 | 5 | 4 | 46 | 39 | 102 |
|  | 347534971 | *Flavobacterium branchiophilum FL-15* | 0 | 3 | 1 | 0 | 1 | 437 | 0 | 2 | 444 |
|  | 261854630 | *Halothiobacillus neapolitanus c2* | 473 | 0 | 0 | 0 | 50 | 2 | 0 | 0 | 525 |
|  | 300112745 | *Nitrosococcus watsoni C-113* | 0 | 0 | 0 | 0 | 1 | 0 | 0 | 0 | 1 |
|  | 327384027 | *Lactobacillus casei BD-II* | 7 | 1 | 0 | 2 | 160 | 1 | 0 | 1 | 172 |
|  | 325278757 | *Odoribacter splanchnicus DSM 20712* | 0 | 0 | 0 | 0 | 2 | 1 | 0 | 2 | 5 |
|  | 111017022 | *Rhodococcus jostii RHA1* | 1 | 0 | 0 | 0 | 2 | 0 | 0 | 0 | 3 |
|  | 182682970 | *Streptococcus pneumoniae CGSP14* | 0 | 0 | 0 | 0 | 4 | 0 | 0 | 0 | 4 |
|  | 307723218 | *Thermoanaerobacter sp. X513* | 0 | 0 | 0 | 0 | 0 | 2 | 0 | 0 | 2 |
|  | 154243958 | *Xanthobacter autotrophicus Py2* | 2 | 0 | 0 | 0 | 0 | 0 | 0 | 0 | 2 |
| **SCIMM**  $\boldsymbol{+d}_{\boldsymbol{2}}^{\boldsymbol{s}}\mathbf{Bin}$ | 256653503 | *Acetobacter pasteurianus IFO 3283-12 DNA* | 8 | 0 | 2 | 0 | 1 | 4 | 38 | 49 | 102 |
|  | 347534971 | *Flavobacterium branchiophilum FL-15* | 0 | 6 | 10 | 0 | 0 | 428 | 0 | 0 | 444 |
|  | 261854630 | *Halothiobacillus neapolitanus c2* | 494 | 1 | 2 | 0 | 24 | 2 | 0 | 2 | 525 |
|  | 300112745 | *Nitrosococcus watsoni C-113* | 0 | 0 | 1 | 0 | 0 | 0 | 0 | 0 | 1 |
|  | 327384027 | *Lactobacillus casei BD-II* | 7 | 1 | 8 | 5 | 150 | 1 | 0 | 0 | 172 |
|  | 325278757 | *Odoribacter splanchnicus DSM 20712* | 0 | 0 | 4 | 0 | 0 | 0 | 0 | 1 | 5 |
|  | 111017022 | *Rhodococcus jostii RHA1* | 1 | 1 | 1 | 0 | 0 | 0 | 0 | 0 | 3 |
|  | 182682970 | *Streptococcus pneumoniae CGSP14* | 0 | 1 | 2 | 0 | 0 | 1 | 0 | 0 | 4 |
|  | 307723218 | *Thermoanaerobacter sp. X513* | 0 | 0 | 1 | 1 | 0 | 0 | 0 | 0 | 2 |
|  | 154243958 | *Xanthobacter autotrophicus Py2* | 2 | 0 | 0 | 0 | 0 | 0 | 0 | 0 | 2 |

**TableS2-5**

| **Tool** | **NCBI Genome ID** | **Genome Name** | **Bin** | | | | | **Number of contigs belong to the genome** |
| --- | --- | --- | --- | --- | --- | --- | --- | --- |
|  |  |  | **1** | **2** | **3** | **4** | **5** |  |
| **MyCC** | 256653503 | *Acetobacter pasteurianus IFO 3283-12 DNA* | 29 | 3 | 52 | 8 | 10 | 102 |
|  | 347534971 | *Flavobacterium branchiophilum FL-15* | 0 | 444 | 0 | 0 | 0 | 444 |
|  | 261854630 | *Halothiobacillus neapolitanus c2* | 209 | 3 | 0 | 313 | 0 | 525 |
|  | 300112745 | *Nitrosococcus watsoni C-113* | 1 | 0 | 0 | 0 | 0 | 1 |
|  | 327384027 | *Lactobacillus casei BD-II* | 143 | 22 | 0 | 7 | 0 | 172 |
|  | 325278757 | *Odoribacter splanchnicus DSM 20712* | 2 | 2 | 0 | 1 | 0 | 5 |
|  | 111017022 | *Rhodococcus jostii RHA1* | 2 | 0 | 0 | 1 | 0 | 3 |
|  | 182682970 | *Streptococcus pneumoniae CGSP14* | 2 | 2 | 0 | 0 | 0 | 4 |
|  | 307723218 | *Thermoanaerobacter sp. X513* | 0 | 2 | 0 | 0 | 0 | 2 |
|  | 154243958 | *Xanthobacter autotrophicus Py2* | 0 | 0 | 0 | 2 | 0 | 2 |
| **MyCC**  $\boldsymbol{+d}_{\boldsymbol{2}}^{\boldsymbol{s}}\mathbf{Bin}$ | 256653503 | *Acetobacter pasteurianus IFO 3283-12 DNA* | 4 | 8 | 69 | 12 | 9 | 102 |
|  | 347534971 | *Flavobacterium branchiophilum FL-15* | 0 | 444 | 0 | 0 | 0 | 444 |
|  | 261854630 | *Halothiobacillus neapolitanus c2* | 38 | 3 | 0 | 484 | 0 | 525 |
|  | 300112745 | *Nitrosococcus watsoni C-113* | 0 | 1 | 0 | 0 | 0 | 1 |
|  | 327384027 | *Lactobacillus casei BD-II* | 163 | 1 | 0 | 8 | 0 | 172 |
|  | 325278757 | *Odoribacter splanchnicus DSM 20712* | 3 | 2 | 0 | 0 | 0 | 5 |
|  | 111017022 | *Rhodococcus jostii RHA1* | 0 | 2 | 0 | 1 | 0 | 3 |
|  | 182682970 | *Streptococcus pneumoniae CGSP14* | 1 | 3 | 0 | 0 | 0 | 4 |
|  | 307723218 | *Thermoanaerobacter sp. X513* | 0 | 2 | 0 | 0 | 0 | 2 |
|  | 154243958 | *Xanthobacter autotrophicus Py2* | 0 | 0 | 0 | 2 | 0 | 2 |
